# Reactive metal-support interaction in the Cu-In_2_O_3_ system: intermetallic compound formation and its consequences for CO_2_-selective methanol steam reforming

**DOI:** 10.1080/14686996.2019.1590127

**Published:** 2019-04-25

**Authors:** Kevin Ploner, Lukas Schlicker, Albert Gili, Aleksander Gurlo, Andrew Doran, Lei Zhang, Marc Armbrüster, Dagmar Obendorf, Johannes Bernardi, Bernhard Klötzer, Simon Penner

**Affiliations:** a Department of Physical Chemistry, University of Innsbruck, Innsbruck, Austria; b Fachgebiet Keramische Werkstoffe/Chair of Advanced Ceramic Materials, Institut für Werkstoffwissenschaften und -technologien, Berlin, Germany; c Advanced Light Source, Lawrence Berkeley National Laboratory, Berkeley, CA, USA; d Faculty of Natural Sciences, Institute of Chemistry, Materials for Innovative Energy Concepts, Chemnitz University of Technology, Chemnitz, Germany; e Institut für Analytische Chemie und Radiochemie, University of Innsbruck, Innsbruck, Austria; f University Service Center for Transmission Electron Microscopy, Vienna, Austria

**Keywords:** Copper, cubic indium oxide, *in situ* X-ray diffraction, Cu_2_In, reduction, 50 Energy Materials, 205 Catalyst / Photocatalyst / Photosynthesis, 212 Surface and interfaces, 503 TEM, STEM, SEM, 504 X-ray / Neutron diffraction and scattering, 106 Metallic materials

## Abstract

The reactive metal-support interaction in the Cu-In_2_O_3_ system and its implications on the CO_2_ selectivity in methanol steam reforming (MSR) have been assessed using nanosized Cu particles on a powdered cubic In_2_O_3_ support. Reduction in hydrogen at 300 °C resulted in the formation of metallic Cu particles on In_2_O_3_. This system already represents a highly CO_2_-selective MSR catalyst with ~93% selectivity, but only 56% methanol conversion and a maximum H_2_ formation rate of 1.3 µmol g_Cu_^−1^ s^−1^. After reduction at 400 °C, the system enters an In_2_O_3_-supported intermetallic compound state with Cu_2_In as the majority phase. Cu_2_In exhibits markedly different self-activating properties at equally pronounced CO_2_ selectivities between 92% and 94%. A methanol conversion improvement from roughly 64% to 84% accompanied by an increase in the maximum hydrogen formation rate from 1.8 to 3.8 µmol g_Cu_^−1^ s^−1^ has been observed from the first to the fourth consecutive runs. The presented results directly show the prospective properties of a new class of Cu-based intermetallic materials, beneficially combining the MSR properties of the catalyst’s constituents Cu and In_2_O_3_. In essence, the results also open up the pathway to in-depth development of potentially CO_2_-selective bulk intermetallic Cu-In compounds with well-defined stoichiometry in MSR.

## Graphical abstract

**Figure:**
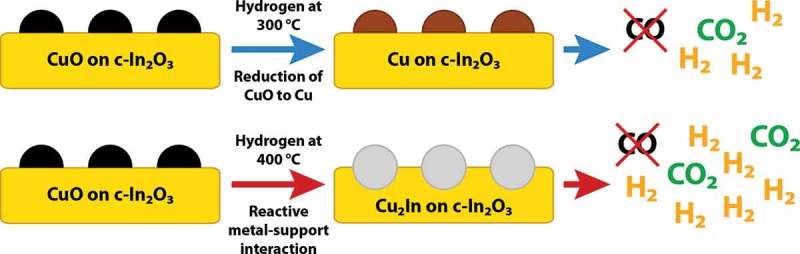

## 1. Introduction

Methanol steam reforming (MSR) remains one of the most important reactions in hydrogen economy to access large amounts of hydrogen that can subsequently be used as a renewable energy carrier [1]. The most crucial parameter steering the reaction to high H_2_ yields is efficient water activation, which is a prerequisite for high CO_2_ selectivities [2]. Among many materials that can be used to selectively catalyze this reaction in the desired direction [3], intermetallic compounds have arisen as a promising material class [4–10]. Pd-based intermetallic compounds have been particularly scrutinized in this respect [11–14], but Cu-Zr materials have also been a focus of research [2,15,16]. As the common denominator to explain the catalytic selectivity patterns, a bifunctional synergistic action of intermetallic compound and supporting oxide, sharing the sites of methanol and water activation, respectively, has been proposed [11,17]. Different synthesis pathways have been reported to gain access to these materials, including reactive metal-support interaction starting from small metal particles on oxide supports (i.e. reduction in hydrogen), yielding intermetallic compound particles distributed on the oxide support and a defined interface between them [5]. ZnPd-ZnO [12], Ga_2_Pd-Ga_2_O_3_ [13] and InPd-In_2_O_3_ [11] are prime examples of such systems. A second pathway is related to the *in situ* decomposition of nanosized or bulk intermetallic compounds in the MSR mixture, yielding both bifunctionally operating interfaces. In this respect, oxidative segregation effects either yield small patches of oxide on the intermetallic compound surface (as observed, for example, for ZnPd/ZnO) [17,18] or a complete corrosive decomposition of the structure into a dispersed metal/oxide system (as prevalent, for example, for Cu_51_Zr_14_) [15,16].

Explaining the catalytic action of such systems necessarily involves a deep understanding of the individual constituents of the material and, in particular, the oxide phase. Depending on the intrinsic catalytic properties, the oxide could beneficially or detrimentally contribute to the catalytic properties of the entire system. As for the latter, selectivity-spoiling effects by the (inverse) water-gas shift equilibrium (e.g. for Ga_2_O_3_) could be observed [11]. However, In_2_O_3_ is the archetypical beneficially acting oxide entity, as it is not only a highly CO_2_-selective (yet comparably inactive) catalytic material in MSR by itself [19] but has recently also been reported to act as a superior catalyst for the methanol synthesis by CO_2_ hydrogenation [20].

As copper is a recurrent metal constituent of many prospective MSR catalysts (despite the ongoing discussion about the nature of the active site, if either strain phenomena or ionic copper species are responsible for the catalytic action) [3], it is straightforward to combine Cu and In_2_O_3_ to potentially create a new MSR catalyst with superior activity and selectivity. Our interest in the system Cu-In_2_O_3_ is also fueled by recent reports on the beneficial influence of Cu on In_2_O_3_ in the formation of methanol by hydrogen/water reduction of CO_2_ [21]. Whether In_2_O_3_ is able to stabilize the potentially active and selective copper species (thereby suppressing the notorious sintering of Cu particles, for example, deactivating Cu particles on ZnO and significantly decreasing the lifetime of these catalysts) or rather leads to specific intermetallic Cu-In particles supported on In_2_O_3_ is in the focus of this article. The Cu-In phase diagram is well known and features a variety of different intermetallic compounds with variable stoichiometries [22]. As the region around 30 at% In exhibits compounds with high melting points, chances are high that single-phase oxide-supported intermetallic compounds can be accessed by reactive metal-support interaction. Additionally, a high temperature Cu_3_In phase, which undergoes eutectoid decomposition upon cooling, was reported to catalyze the oxidative dehydrogenation of methanol to formaldehyde. This proves that the obtained eutectic cast with the overall stoichiometry Cu_3_In is able to activate methanol, even though the resulting phases are not identified by the authors in [23].

This article focuses on two selected research areas, whose comprehension is necessary to develop a knowledge-driven synthesis routine and enable a full understanding of the operation of the Cu-In_2_O_3_ system in MSR. First, the reactive metal-support interaction between Cu and In_2_O_3_, that is, the reduction in hydrogen and the corresponding intermetallic compound formation, will be assessed. As the removal of reaction-induced water is a key parameter in the efficient formation of intermetallic compounds, we will perform experiments under recirculating batch and quasi-flowing conditions. This potentially enables us to access different oxide-supported single-phase intermetallic Cu-In compounds, whose catalytic properties can then be assessed. In the second part, we test the properties of these oxide-supported intermetallic Cu-In compound particles in MSR and relate the results to previously tested intermetallic materials, especially systems based on Pd. An integral part of the characterization will be devoted to synchrotron-based *in situ* X-ray diffraction (XRD) measurements to follow the structural transitions in the course of reactive metal-support interaction.

## 2. Experimental details

### 2.1. Preparation of the Cu/c-In_2_O_3_ catalyst

Bixbyite-type cubic indium oxide (c-In_2_O_3_, space group *Ia*$\overset{ˉ}{3}$, *a* = 10.118 Å [24]; Alfa Aesar, 99.99% metals basis) was suspended in deionized water and an aqueous solution of Cu(II) acetate (Cu(OOCCH_3_)_2_**·**H_2_O; Merck) was added dropwise under stirring. The solvent was subsequently removed under vacuum at 60 °C and the resulting turquoise powder calcined in air at 400 °C for 2 h, yielding the gray CuO/c-In_2_O_3_ catalyst with a nominal loading of 8.0 wt% CuO in the calcined state (corresponding to 6.5 wt% metallic Cu after pre-reduction).

### 2.2. Catalytic measurements

Catalytic testing was performed in a recirculating batch reactor connected to a quadrupole mass spectrometer (QMS) arranged in cross-beam geometry, equipped with a secondary electron multiplier (Balzers QMG 311). This setup is specialized for the measurement of small sample amounts (approximately 100 mg) and conversions (reactor volume = 13.8 ml). The reactor and the sample holder are made of quartz glass and can be heated up to 1100 °C in a Linn High Term furnace. The temperature is monitored with a K-type thermocouple (NiCr-Ni).

For the MSR measurements, ~28 mbar of a mixture with a ratio of methanol:water = 1:2 (v/v) in the gas phase is prepared to avoid water depletion during the reaction. One MSR cycle consists of three steps: (1) pre-oxidation at 400 °C in 1 bar pure O_2_ for 1 h (termed O400), (2) pre-reduction at 300 °C in 1 bar pure H_2_ for 1 h (termed H300) and (3) MSR reaction. The latter is carried out by the addition of Ar to the MSR mixture (for correction of the signals considering the thermal expansion and the capillary leak to the QMS) and balancing the pressure to 1 bar with He (for improvement of the recirculation efficiency and the thermal conductivity) at 100 °C. Starting from this temperature, a heating ramp with 5 °C min^−^^1^ is applied, and the gas phase composition is continuously monitored by the QMS.

The formation rates are obtained by differentiation of the partial pressure versus reaction time graphs, which is acquired by the application of the calibration. Then, the conversion to μmol using the ideal gas law and normalization to the total copper mass determined by differential pulse voltammetry (DPP; see sections 2.4 and 3.1) yields the formation rates in μmol gCu^−1^ s^−1^.

The apparent activation energy for the CO_2_ evolution (*E*_a_) was calculated by Arrhenius fitting of the formation rate versus the temperature plot at the beginning of the rate increase, thus, excluding influences of the reaction products on the equilibrium. To enhance the comparability of the apparent activation energy between different catalytic materials, the pre-exponential factor *A* is fixed to a value of 10^8^ µmol g_Cu_^−1^ s^−1^ to avoid any influence of statistical fluctuations on the fitting procedure. These values are in good agreement with pre-exponential factors reported by Baetzold and Somorjai for diffusion-controlled surface reactions [25].

### 2.3. Structural characterization

The *ex situ* XRD measurements were performed in the transmission mode utilizing a Stadi P diffractometer (STOE & Cie GmbH, Darmstadt, Germany). This setup is equipped with a MYTHEN2 DCS4 detector (DECTRIS Ltd., Switzerland) and a Mo X-ray tube (GE Sensing & Inspection Technologies GmbH, Ahrensburg, Germany) with a heating current of 40 mA and an acceleration voltage of 50 kV. A curved Ge(111) crystal selects the Mo K_α1_ radiation with a wavelength of 0.7093 Å. The analysis of the diffractograms was carried out with the software WinX^POW^ using reference data from the ICDD database [26,27].^⁠^

The *in situ* high-temperature synchrotron XRD experiments in H_2_ were performed at the beamline 12.2.2, Advanced Light Source (ALS), Lawrence Berkeley National Lab, CA, USA. The *in situ* diffraction patterns were collected in the angle-dispersive transmission mode with a focused 25-keV monochromatic beam (λ = 0.4984 Å, 30 μm spot size). The sample powder was heated in a 0.7-mm quartz capillary under a continuous gas flow (10 ml min^−1^ pure H_2_) injected through a 0.5-mm tungsten tube. The capillary is heated at 10 °C min^−1^ to 500 °C in an infrared-heated *SiC* tube furnace as described in [28,29]. Diffraction patterns were recorded by a PerkinElmer flat panel detector (XRD 1621, dark image and strain correction) every 35 s during the heating cycle.

Transmission electron microscopy (TEM) measurements were carried out using a FEI Tecnai F20 S-TWIN analytical (high-resolution) transmission electron microscope (200 kV), equipped with an Apollo XLTW SDD X-ray detector (for collecting energy-dispersive X-ray (EDX) data).

### 2.4. DPP

The total copper content of the CuO/c-In_2_O_3_ catalyst was determined by DPP) in 0.05 M sodium acetate as supporting electrolyte. Measurements were performed on a CV 50 W potentiostat (BAS) controlled by the software CV 50 (BAS) in combination with an EG&G 303a electrode stand and a classical three-electrode configuration with a hanging mercury drop electrode (HMDE; drop size M) as working electrode, a platinum wire auxiliary electrode and a Ag/AgCl/3 M KCl reference electrode. A pulse amplitude of 50 mV with a duration of 50 ms, a sampling time of 20 ms, a pulse period of 200 ms and a scan rate of 10 mV s^−1^ was applied during each voltammetric scan. The Cu^2+^/Cu reduction peak occurs at 0.050 V versus Ag/AgCl/KCl (3 M) under these measurement conditions. Removal of oxygen from the solution is essential and was achieved by purging at least for 10 min with nitrogen 5.0 before the first recording of the voltammogram of the pure supporting electrolyte and at least 2 min after each sample or standard addition.

## 3. Results and discussion

### 3.1 Determination of the copper content of Cu/c-In_2_O_3_ by DPP

Two different sample solutions containing nominal concentrations of 61 ppm Cu and 5.8 ppm Cu were analyzed. Quantification of the total copper amount was performed by standard addition and external calibration using freshly prepared 100 and 10 ppm Cu standard solutions. The more concentrated sample contained 49.7 mg CuO/c-In_2_O_3_ with about 6.4 wt% copper dissolved in 50 ml of about 7% HNO_3_. For this sample, an average total amount of 48 ppm Cu was obtained by DPP. For the diluted sample of 9.4 mg CuO/c-In_2_O_3_ with the same nominal copper content dissolved in 100 ml 7% HNO_3_, an average amount of 4.65 ppm Cu was found by DPP. Since both samples contain the same Cu/c-In_2_O_3_ catalyst, an average copper loading of 6.4 wt% CuO and 5.1 wt% Cu in the calcined state can be deduced.

### 3.2 Reactive metal-support interaction between Cu and In_2_O_3_: monitoring the intermetallic compound formation between Cu and In during reduction in hydrogen

The formation of intermetallic Cu-In compounds was investigated at first by *ex situ* powder XRD after different pre-reduction temperatures in the recirculating batch reactor to directly relate the structural consequences during pre-reduction to the subsequent catalytic measurements in MSR. A measure of 1 bar of pure H_2_ for pre-reduction was used for 1 h, whereby the temperature was increased stepwise from 100 °C to 500 °C. Higher reduction temperatures were not used, since at 500 °C, the In_2_O_3_ support already starts to be reduced to metallic In. A fresh calcined sample was used for each treatment. The label indicating the last treatment performed on a sample consists of a letter for the respective gas (A for air, O for O_2_, H for H_2_, MSR for the MSR mixture) and a value for the temperature (i.e. H100 denotes pre-reduction in pure H_2_ at 100 °C for 1 h).

**Figure 1.**
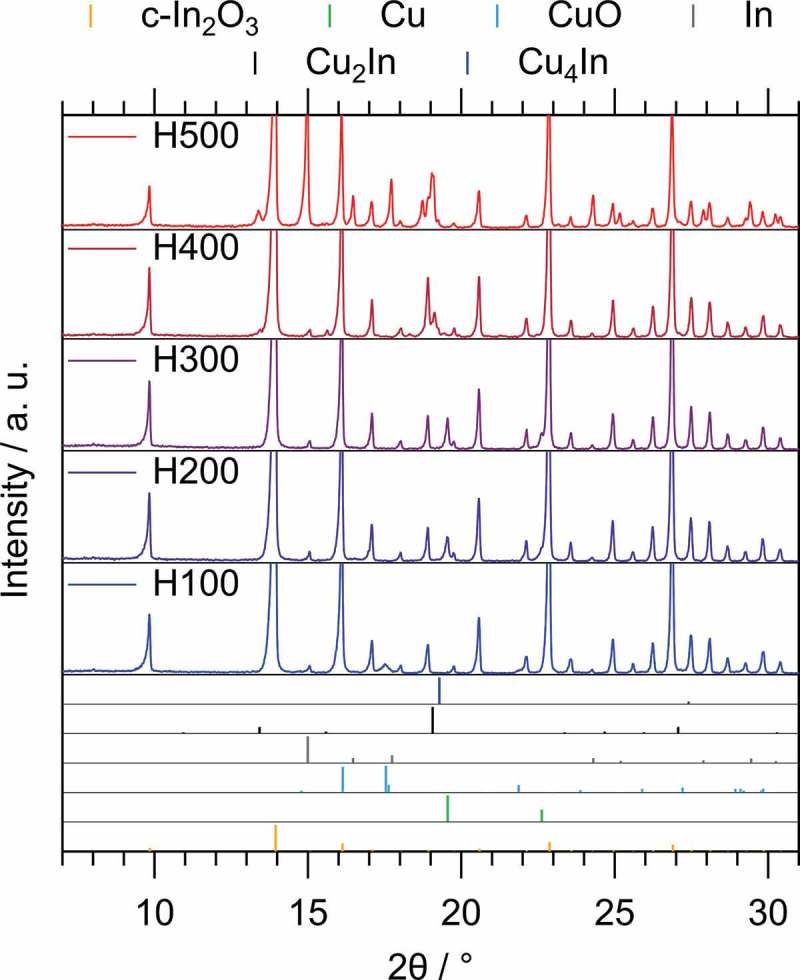
*Ex situ* XRD diffraction patterns collected on the Cu/c-In_2_O_3_ catalyst after pre-reduction in 1 bar pure H_2_ for 1 h at different temperatures from 100 °C (bottom) to 500 °C (top). The most intense reflections have been cut to enhance the visibility of the other peaks. Reference diffractograms (color-coded bars with respective legend on top) have been considered and taken from the ICDD database [27] (PDF files: c-In_2_O_3_ 00-006-0416 [24]; Cu 00-004-0836 [34]; Cu_2_O 01-071-3645 [31] (not observed here); CuO 01-080-1916 [32]; In 03-065-9292 [33]; Cu_2_In 00-042-1475 [30]; Cu_4_In 00-042-1477 [30]).

The *ex situ* collected XRD patterns of the pre-reduction series at five temperatures from 100 °C to 500 °C are displayed in Figure 1. Starting at 100 °C, Cu remains fully oxidized (as CuO), whereas reduction at 200 °C already yields metallic Cu on the yet unchanged c-In_2_O_3_ support. The H300 sample appears structurally very similar to the one treated at 200 °C, but a further increase to 400 °C leads to the formation of intermetallic Cu-In compounds. According to the Cu-In phase diagram, Cu_2_In is the intermetallic phase with the highest melting point. The most prominent reflections in the H400 XRD pattern can be assigned to such a hexagonal Cu_2_In structure with the space group *P*6_3_/*mmc* (*a* = 4.2943 Å and *c* = 5.2328 Å) [30]. Cu_4_In could also be present, as it also displays its most intense reflection in the same 2*θ* region. However, the other reflections do not clearly match Cu_4_In. Metallic copper vanishes completely, which further corroborates the formation of intermetallic Cu-In compounds. Most of the Cu-In intermetallic reflections are somewhat shifted from their ideal position, which can be explained by the still ongoing phase formation and the homogeneity range, allowing for the incorporation of some amounts of Cu/In into the parent intermetallic compound lattice resulting in deviations from the ideal lattice parameters.

After pre-reduction at 500 °C, c-In_2_O_3_ starts to become partially reduced to metallic In, and the reflections of the intermetallic compounds change significantly in their position, as well as in their relative intensity. The shift in the diffraction angle could once more be the result of a phase width of a distinct intermetallic Cu-In compound with a concomitant change of the lattice parameters. In summary, we can conclude that after a reduction treatment at 300 °C, metallic Cu, but no bulk intermetallic Cu-In, compound is present, whereas after reduction at 400 °C, the metallic Cu state disappears and is replaced by Cu_2_In.

**Figure 2.**
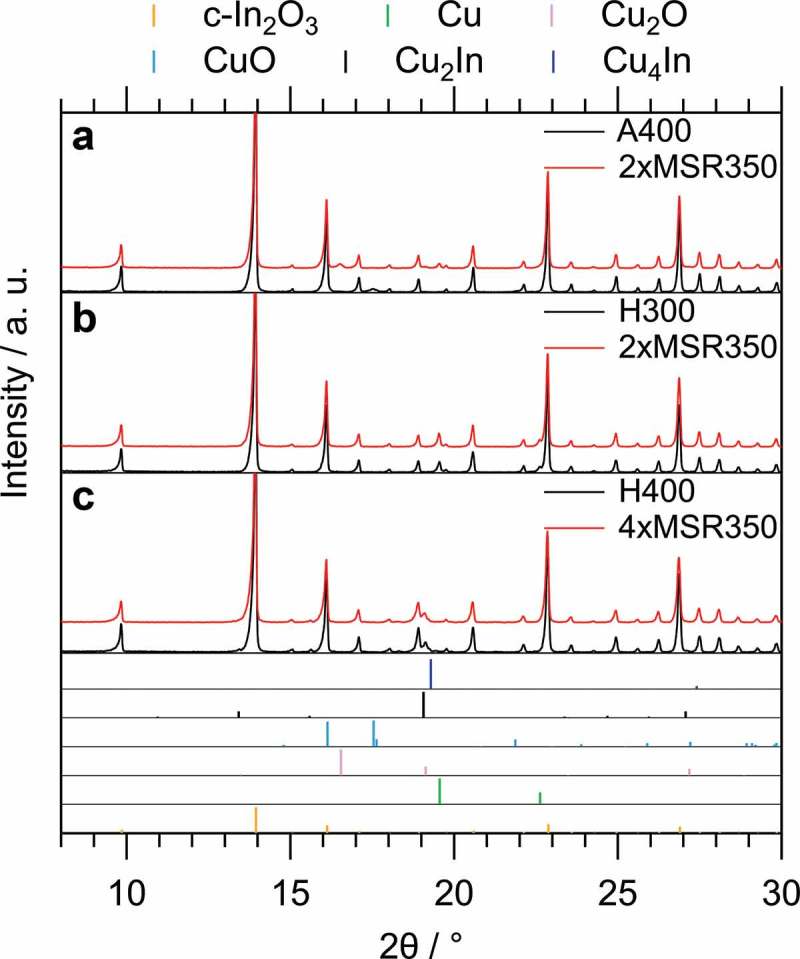
Comparison of the *ex situ* collected XRD patterns of the Cu/c-In_2_O_3_ catalyst before and after MSR using different pre-treatments. The most intense reflections have been cut to increase the visibility of the other peaks. The diffractograms after MSR are depicted with an offset. The references were taken from the ICDD database [27] (for PDF numbers, see caption of Figure 1). Panel A shows the XRD patterns after calcination (A400), O400 and two MSR cycles without pre-reduction; Panel B compares the patterns after O400, H300 and two MSR cycles; and Panel C compares the patterns after O400, H400 and four MSR cycles.

We mention already at this stage that a pre-reduction at 300 °C and 400 °C conveniently allows to assess the potential influence of intermetallic compound formation on the catalytic behavior in MSR as a test reaction. Two states can, hence, directly be accessed: Cu/c-In_2_O_3_ (300 °C) and Cu_2_In/c-In_2_O_3_ (400 °C). An obvious prerequisite, however, is the bulk structural stability of the phases during the catalytic reaction. This has been assessed by *ex situ* XRD and is shown accordingly in Figure 2. Starting from the calcined state (A400, with an additional O400 treatment prior to MSR, ensuring that the sample is completely oxidized and removing residual surface impurities), the catalyst is reduced to a mixture of Cu_2_O and Cu by the MSR mixture in the course of reaction (diffractograms after two MSR cycles each consisting of O400 and MSR up to 350 °C (termed MSR350) are being depicted in Panel A of Figure 2). Introducing an additional pre-reduction step at 300 °C in H_2_ before MSR changes the initial state to fully reduced Cu on c-In_2_O_3_ and no significant changes in the diffractogram are visible after two complete MSR cycles (each consisting of O400, H300 and MSR350, see Figure 2, Panel B). Also after raising the pre-reduction temperature to 400 °C, only minor alterations in the reflections assigned to the intermetallic Cu_2_In compound are observed, although four complete MSR cycles were conducted.

**Figure 3.**
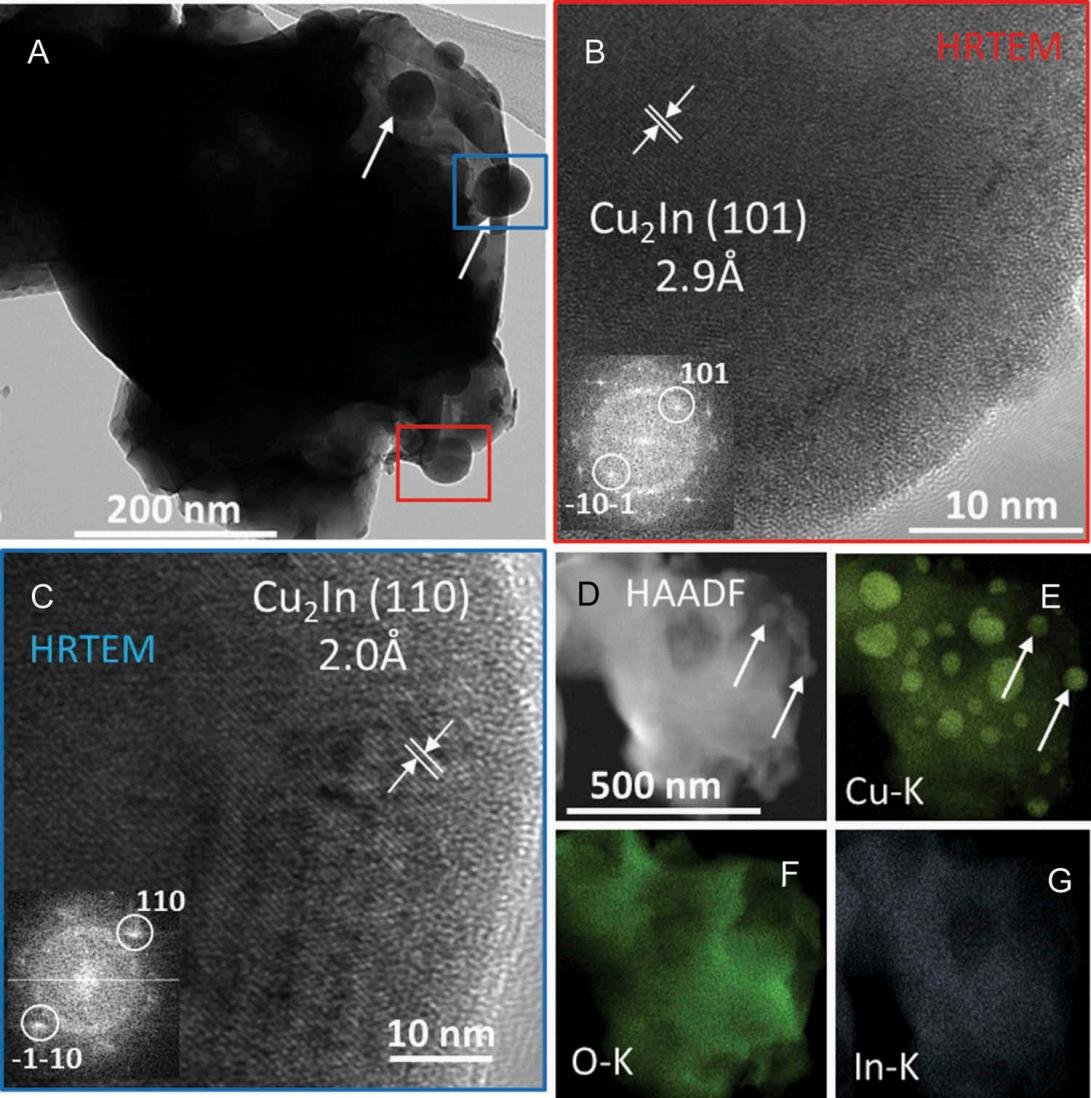
Transmission electron microscopy analysis of the intermetallic compound state of the Cu/c-In_2_O_3_ system after reduction in hydrogen at 400 °C for 1 h. Panel A: bright field overview image. The red and blue boxes indicate the particles that are analyzed by high resolution microscopy as highlighted in Panels B and C. Panels B and C: high resolution images of two representative Cu_2_In particles exhibiting (101) and (110) lattice fringes, respectively. The fast Fourier transforms are shown as insets. Panels D–G: EDX analysis of the area shown in Panel A. HAADF image (D) and the individual elemental maps of Cu-K (Panel E), O-K (Panel F) as well as In-K (Panel G) are also shown.

**Figure 4.**
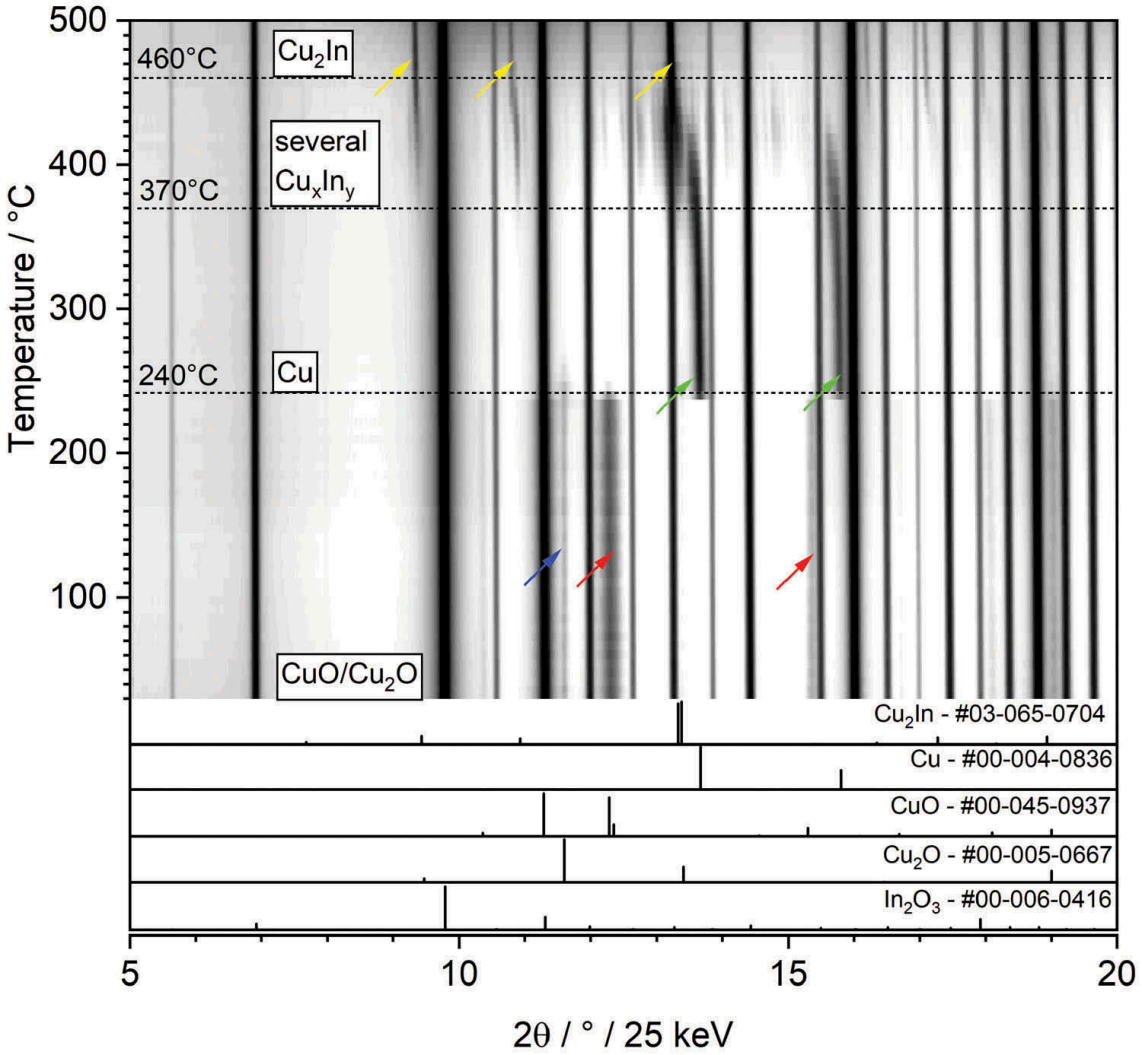
Contour plot of the *in situ* X-ray diffraction analysis following the treatment of the Cu/c-In_2_O_3_ catalyst in pure H_2_. The 2*θ* range 5–25° is shown in order to follow the transition from CuO with some Cu_2_O over metallic Cu to Cu_2_In. The main reflections for these structures are marked with colored arrows (CuO: red, Cu_2_O: blue, Cu: green, Cu_2_In: yellow) [24,27,34–37]. Note that the reaction scenario outlined in Equation (1) is not the only pathway that might lead to intermetallic compound formation. In terms of structural consequences of reactive metal-support interaction, the main question remains, if (i) first oxidized Cu is being reduced to metallic Cu, then In_2_O_3_ reduction to metallic In takes place and subsequently In reacts with Cu to form Cu_x_In intermetalloids, (ii) if it proceeds as outlined in the reaction according to Equation (1) or (iii) CuO and c-In_2_O_3_ are reduced simultaneously to form Cu_x_In phases. So far, the data suggest that reduction of both oxidized Cu and In_2_O_3_ occurs, but thermodynamic considerations, especially also with respect to the relative stabilities of the participating phases, are needed.

Further insight into the reactive metal-support interaction between Cu and cubic In_2_O_3_ is given by transmission electron microscopy analysis (Figure 3). The bright field overview image in Panel A clearly shows the decoration of an In_2_O_3_ grain with several small particles with rounded outlines and rather large particle sizes between 30 and 80 nm after a H400 reduction step. Two selected particles have been marked, which are correspondingly also visible in the high-angle annular dark-field (HAADF) image and the Cu-K EDX map of Panel E. Further structural analysis of the two particles in the red and the blue boxes indicate the intermetallic compound formation between Cu and In, following reduction of c-In_2_O_3_ and subsequent interdiffusion of In and Cu. The observed fringes are clearly located at the individual particles and do not extend into the support grains. Lattice fringes of the hexagonal intermetallic Cu_2_In compound measured at ~2 Å and ~3 Å are clearly visible (space group *P*6_3_/*mmc, a* = 4.2943 Å and *c* = 5.2328 Å, *d*_theor_(110) = 2.14 Å, *d*_theor_(101) = 3.03 Å) [30]. The combined structural analysis, hence, indicates the presence of Cu_2_In (also on the basis of the *in situ* reduction studies displayed in Figure 4). On a qualitative basis, the intermetallic compound formation can also be directly inferred from combined HAADF/EDX imaging (Figure 3, Panels D–G). The individual EDX maps of the Cu-K and In-K intensities clearly show the simultaneous presence of Cu and In at the same locations. The Cu particles can be seen clearly, but due to the underlying In_2_O_3_ support, the In-K intensity is more homogeneously distributed. Interestingly, the Cu_2_In particles can also be seen in the HAADF image due to the different average atom number of Cu_2_In and In_2_O_3_. Generally, the HAADF intensity can be approximated as *I*_HAADF_ ≈ *t*·*ρ*·*Z*^1.5^, where *t* is the sample thickness, *ρ* the material density and *Z* the average atom number. Hence, constant thickness and density provided, the HAADF intensity is dominated by *Z*, showing elemental contrast. Areas with higher average atom number, therefore, appear brighter in an HAADF image. Applying this concept to the Cu-In_2_O_3_ system, we note that the average atom number *Z* of In_2_O_3_ is 24.4, the one of Cu 29.0 and the one of Cu_2_In 35.6. Hence, the HAADF intensities *I* of the three components under question can be approximated by 1396*t*, 2042*t* and 858*t* for Cu, Cu_2_In and In_2_O_3_, respectively (assuming densities of 8.936 g cm^−3^ for Cu [34], 9.614 g cm^−3^ for Cu_2_In [30] and 7.117 g cm^−3^ for In_2_O_3_ [24]). The intensity difference between the intermetallic compound and In_2_O_3_ is the largest, directly validating the observed contrast.

Summarizing the reactive metal-support interaction between Cu and cubic In_2_O_3_, we note that the interaction, as observed for corresponding metal–oxide systems, can be described as a multistep process [5]. The latter may involve reduction of the oxide support species (In_2_O_3_ in this case), followed by diffusion of the reduced In_2_O_3_ species (even In metal) into the Cu lattice and subsequent formation of the intermetallic compound. A tentative reaction of Cu, In_2_O_3_ and H_2_ to result in Cu_2_In is given by

(1) $$8Cu+2In_{2}O_{3}+6H_{2}\to 4Cu_{2}In+6H_{2}O$$

The thermodynamic driving force for intermetallic compound formation is, thus, the efficient removal of reaction-formed water, which shifts the equilibrium toward the products. Thus, it can be expected that a treatment in the recirculating batch reactor, where the water basically is not removed, merely causes a minor reduction of In_2_O_3_. Hence, also the intermetallic compound formation is apparently less efficient compared to treatments, where water is constantly removed. To assess the influence of water removal, *in situ* XRD measurements have been performed. In this capillary setup, quasi-flowing conditions can be realized as discussed in the experimental section. These are collectively shown in Figure 4.

In the course of the experiment, qualitatively the same sequence of phase transformations can be observed, corroborating the data shown in Figure 1. Starting from oxidized Cu particles on c-In_2_O_3_, formation of metallic Cu sets in at around 240 °C, before reduction of In_2_O_3_ and intermixing of Cu and In is observed roughly between 370 °C and 450 °C. In this temperature region, a complex phase mixture of several intermetallic Cu-In compounds is observed, before finally at 460 °C, Cu_2_In prevails as the sole intermetallic compound. The 2*θ* shift in the reflections of Cu_2_In and Cu in the temperature range of Cu_2_In formation suggests that the intermetallic compound is formed by interdiffusion of Cu and In. This finding directly corroborates the phase assignment in the *ex situ* XRD measurements, where around 400 °C also Cu_2_In prevails as the main phase.

### 3.3 Influence of the intermetallic compound formation on the CO_2_ selectivity and performance in MSR

**Figure 5.**
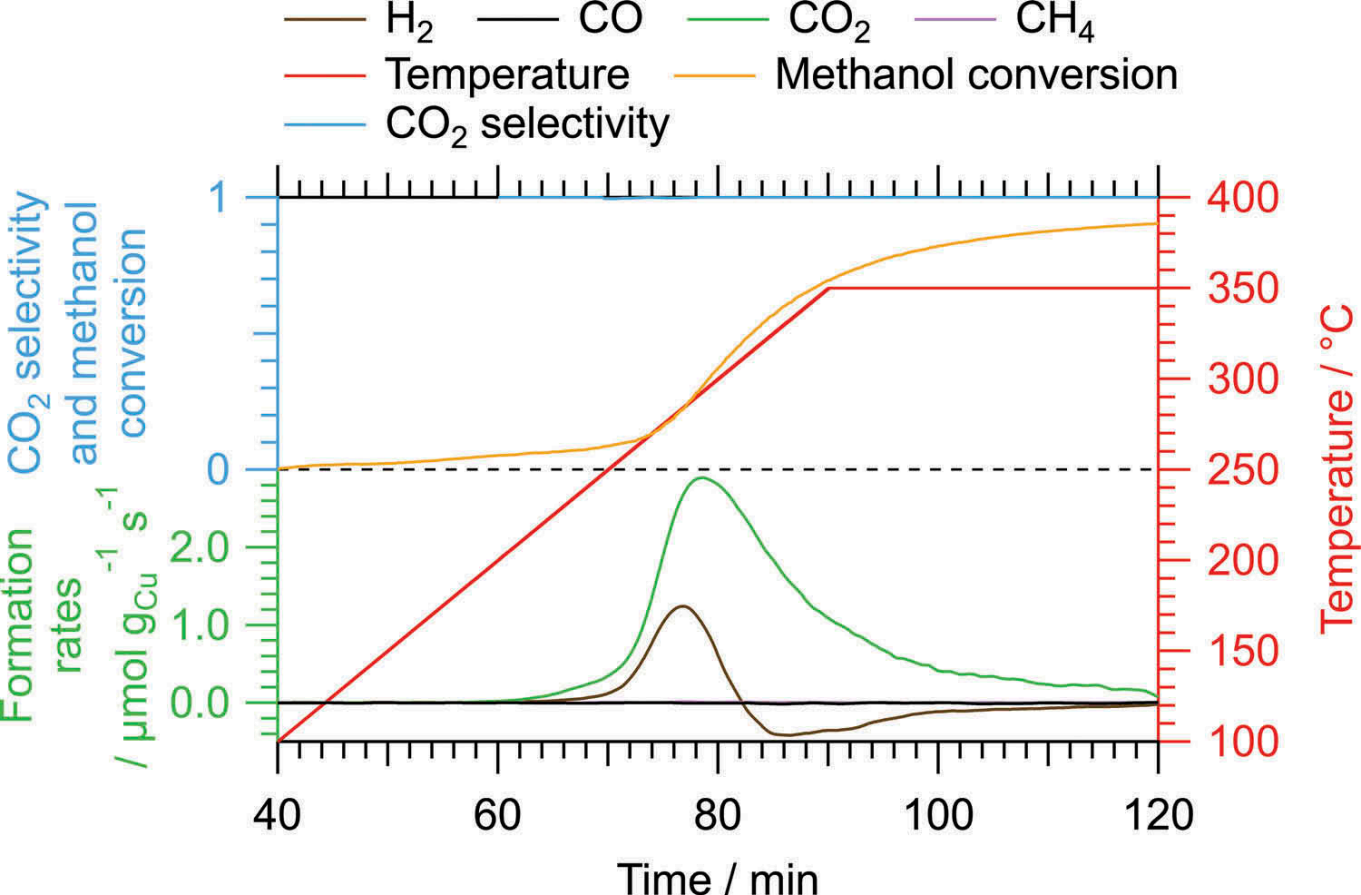
Catalytic profile of one MSR cycle after oxidation in pure oxygen at 400 °C (O400) without further pre-reduction (starting from CuO/c-In_2_O_3_). The formation rates (in µmol g_Cu_^−1^ s^−1^; green axis) of H_2_, CO, CO_2_ and CH_4_; the CO_2_ selectivity (blue axis and trace); and the methanol conversion (blue axis, but orange trace) are plotted versus the reaction time. The temperature profile is depicted in red.

The influence of intermetallic compound formation starting from CuO on c-In_2_O_3_ on the performance in MSR was investigated by starting the reaction from different initial states. The most important catalytic parameters of relevant cycles of the three initial states are summarized in Table 1. In Figure 5, the MSR reaction was conducted after calcination with an additional oxidation step at 400 °C for 1 h in pure O_2_ (O400). The formation of hydrogen and carbon dioxide starts at approximately 180 °C, but no CO and only traces of CH_4_ (below 0.05 mbar in total) are produced. As expected, hydrogen evolving in the beginning is almost entirely consumed again, reducing the initially present CuO to Cu_2_O and Cu (see also Figure 2, Panel A). The methanol conversion reaches a maximum of 90%, and the CO_2_ selectivity amounts to 100% at any time because of the absence of CO formation. The decrease in the formation rates in all catalytic profiles is caused by the depletion of the reaction mixture, which is inherent to a batch reactor. The second cycle on this system was omitted, because it exhibits an almost identical behavior.

**Table 1.** Summary of key catalytic parameters of the methanol steam reforming reaction starting from pre-oxidized CuO (O400), from metallic Cu (H300) and from Cu_2_In (H400) on c-In_2_O_3._

	O400 CuO/c-In_2_O_3_	H300 Cu/c-In_2_O_3_	H400 (cycle 1) Cu_2_In/c-In_2_O_3_	H400 (cycle 4) Cu_2_In/c-In_2_O_3_
Light-off *T*(H_2_) (°C)	180	200	230	230
Light-off *T*(CO) (°C)	–	290	300	300
Light-off *T*(CO_2_) (°C)	180	200	230	230
Light-off *T*(CH_4_) (°C)	270	310	320	300
Maximum rate H_2_ (µmol g_Cu_^−1^ s^−1^)	1.2	1.3	1.8	3.8
Maximum rate CO (µmol g_Cu_^−1^ s^−1^)	–	0.02	0.02	0.07
Maximum rate CO_2_ (µmol g_Cu_^−1^ s^−1^)	2.9	0.45	0.69	1.3
Maximum rate CH_4_ (µmol g_Cu_^−1^ s^−1^)	0.11	0.02	0.02	0.01
Final methanol conversion (%)	90	56	62	84
Minimum CO_2_ selectivity (%)	100	93	94	92
*E*_a_(CO_2_) (kJ mol^−1^)	–	101	99	97

**Figure 6.**
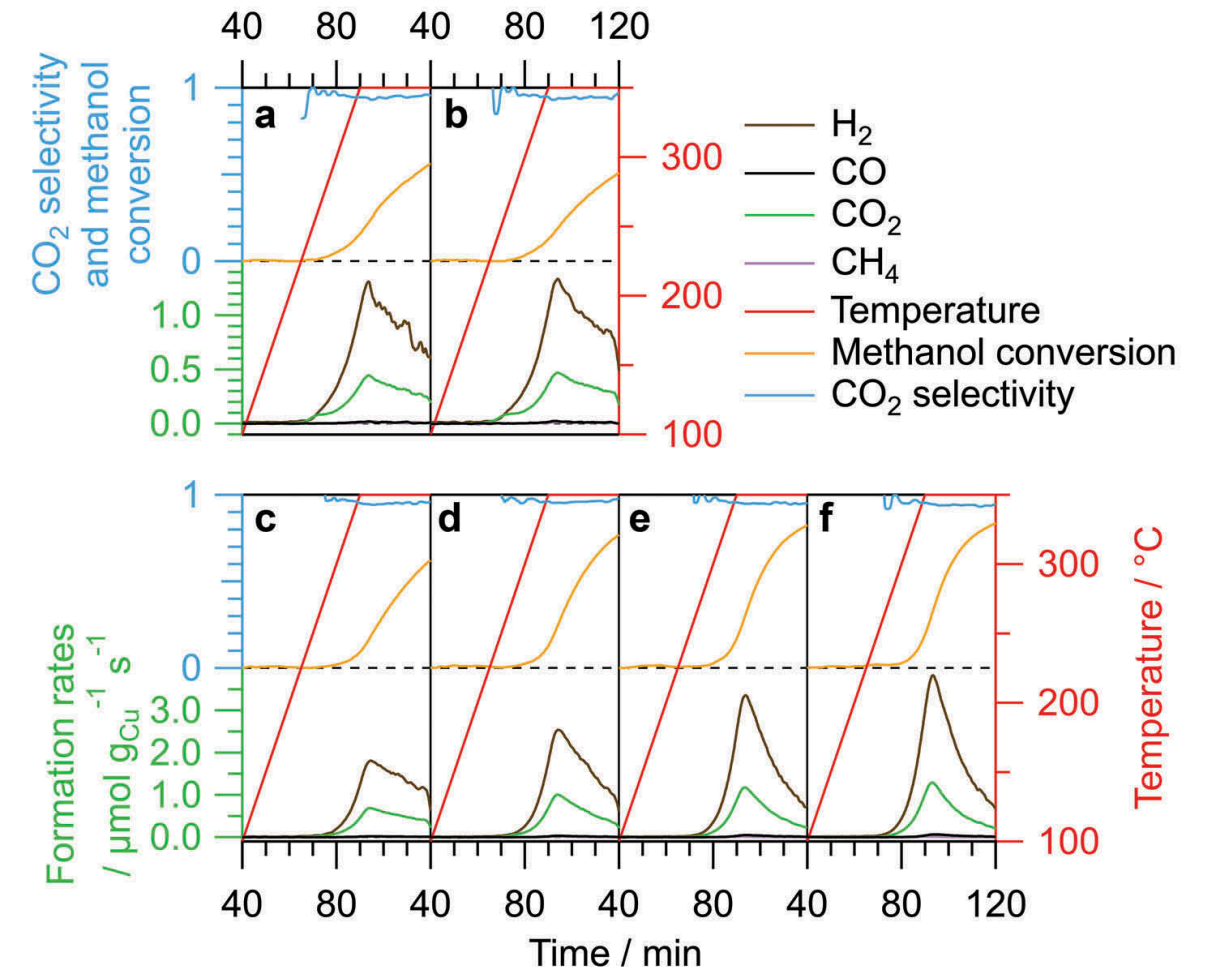
Catalytic profiles of two MSR cycles with O400 and H300 (starting from Cu/c-In_2_O_3_; Panels A and B) and four MSR cycles with O400 and H400 (starting from Cu_2_In/c-In_2_O_3_; Panels C–F). In each graph, the formation rates (in µmol g_Cu_^−1^ s^−1^, green axis) of H_2_, CO, CO_2_ and CH_4_; the CO_2_ selectivity (blue axis and trace); and the methanol conversion (blue axis, but orange trace) are plotted versus the reaction time. The temperature profile is depicted in red.

Starting MSR after pre-reduction in 1 bar pure H_2_ for 1 h at 300 °C yields the catalytic profiles depicted in Panels A and B of Figure 6, which represent two full catalytic cycles (O400, H300, MSR350) on the same sample without exposure to air. In contrast to the measurements without pre-reduction, hydrogen is formed as the main product of the reforming reaction with a rate that is three times as high at its maximum as the corresponding value for CO_2_ at the same time, representing the ideal stoichiometry of MSR. Only traces of CO and CH_4_ are being formed with their maximum rates amounting to approximately 1.5% (1.8% CO and 1.0% CH_4_ in the second cycle) compared to H_2_. This is also reflected in the CO_2_ selectivity, which exceeds 93% over the course of the entire measurement in both cycles. The methanol conversion, however, only reaches a final value of 56% (51% in the second cycle), suggesting a rather low activity of the catalyst.

Increasing the pre-reduction temperature to 400 °C, that is, starting from Cu_2_In/c-In_2_O_3_, leads to the catalytic patterns of four complete MSR cycles (O400, H400, MSR350) depicted in Panels C–F of Figure 6, which look similar to the ones obtained by pre-reduction at 300 °C (see Figure 6, Panels A and B). The differences are the absence of the noncatalytic H_2_ consumption and the overall higher formation rates. Additionally, the activity increases with increasing number of catalytic cycles. Starting from H400 instead of H300 increases the H_2_ formation rate by a factor of 1.5 in the first cycle and more than 3 times in the fourth cycle. Conducting four catalytic cycles on the same catalyst, thus, doubles the H_2_ formation rate, while the initial final methanol conversion of 62% is enhanced to 84% during the fourth cycle. However, the CO formation rate also slightly increases, which leads to a decrease in the minimum CO_2_ selectivity from 94% to 92%, whereas the CH_4_ formation rate is lowered despite the increase in total activity.

Putting these results into perspective of the performance of previous intermetallic compounds in MSR especially on Pd basis, we note several similarities and differences. Speaking of reactive metal-support interaction, intermetallic compound formation in the Pd-In system, starting from Pd-In_2_O_3_, is much more efficient and occurs at much lower temperatures than on Cu-In_2_O_3_ [11,38]. InPd is formed already after reduction at 300 °C, followed by even In-richer intermetallic compounds at higher temperatures. The most intriguing difference between the two systems is the formation of thin TEM-detectable In_2_O_3_ layers around the InPd particles after contact to the MSR reaction mixture in batch reactor experiments, thus deactivating the catalyst after use due to the removal of active intermetallic surface/intermetallic compound-supporting oxide interfacial area. Apparently, this does not happen to this extent for the Cu-In_2_O_3_ material, which further activates after several catalytic cycles in the batch reactor. The immediate reason for this behavior in a very simplified picture might be found in the general lower indium content in the formed intermetallic Cu-In compounds, rendering the decomposition of the latter into a thin oxide layer surrounding the particles less likely. Note that the principal simple formation of a thinner, possibly also amorphous oxide layer, being invisible in XRD or TEM is excluded, as the catalytic profiles are very sensitive to changes in the surface composition. An immediate loss of Cu-In intermetallic surface area would, thus, be catalytically recognized. Comparing the catalytic key parameters between In-Pd and In-Cu catalysts reveals lower activation energies for the former (64 kJ mol^−1^ on InPd/In_2_O_3_ [39] vs. around 96 kJ mol^−1^ for Cu_2_In/c-In_2_O_3_, see Table 1) but similar CO_2_ selectivities (>95% for InPd/In_2_O_3_ [39] vs. >92% for Cu_2_In/c-In_2_O_3_, see Table 1). CO_2_ light-off temperatures are also comparable at around 230–250 °C for both. In combination with the obvious structurally favorable stability of the Cu_2_In/c-In_2_O_3_ interface, it renders the latter a more attractive choice as a CO_2_-selective MSR catalyst.

## 4. Conclusions

In conclusion, we have shown that reactive metal-support interaction in the Cu-In_2_O_3_ system leads to the formation of an intermetallic Cu_2_In compound after reduction in hydrogen at 400 °C. Higher reduction temperatures lead to complex phase mixtures and eventually to the reduction of In_2_O_3_ to In metal. Cu_2_In itself is structurally stable during MSR. Clearly, different catalytic profiles between a Cu/c-In_2_O_3_ and a Cu_2_In/c-In_2_O_3_ interface in MSR with respect to self-activation and general activity have been observed. Therefore, Cu_2_In/c-In_2_O_3_ is found to be superior to Cu/c-In_2_O_3_ in terms of activation at a comparable CO_2_ selectivity, with a doubling of activity from the first run to the fourth run (accompanied by a 20% increase in the methanol conversion) at a constantly high CO_2_ selectivity between 92% and 94%. This article, hence, features a new prospective class of intermetallic compound-based highly CO_2_-selective MSR catalysts using c-In_2_O_3_ as starting support material before exploiting reactive metal-support interaction to access the intermetallic compounds. As especially in the *in situ* XRD measurements plenty of different intermetallic Cu-In phases have been observed after reduction in a temperature segment close to where Cu_2_In has been obtained, future studies must be directed at the deliberate synthesis of intermetallic Cu-In compounds with controlled and defined stoichiometry to enable the knowledge-driven assessment of the ideal Cu-In ratio and the corresponding oxidative segregation/formation tendency of a potentially CO_2_-selective intermetallic Cu-In compound/c-In_2_O_3_ interface. In particular, it needs to be assessed whether this segregation occurs at all (i.e. to answer the question if the interface is actually required) or whether the intermetallic compound itself is able to activate both methanol and water. If such an interface is indeed necessary for a high CO_2_ selectivity, the Cu/c-In_2_O_3_ system can be incorporated into the large group of oxide-supported intermetallic compound catalysts (e.g. Cu_51_Zr_14_/ZrO_2_, ZnPd/ZnO, Ga_2_Pd/Ga_2_O_3_ or InPd/In_2_O_3_), where such a bifunctional synergism of oxide (water activation) and intermetallic compound (methanol activation) is prevalent. The presented results strongly suggest that this is indeed the case.
